# 早期非小细胞肺癌外科治疗新进展

**DOI:** 10.3779/j.issn.1009-3419.2016.06.20

**Published:** 2016-06-20

**Authors:** 坚 胡, 飞潮 包

**Affiliations:** 310003 杭州，浙江大学医学院附属第一医院胸外科 Department of Thoracic Surgery, the First Affiliated Hospital of Zhejiang University Medical College, Hangzhou 310003, China

**Keywords:** 肺肿瘤, 外科, 治疗, Lung neoplasms, Surgery, Treatment

## Abstract

肺癌是恶性肿瘤患者首位的死亡原因，随着计算机断层扫描（computed tomography, CT）等影像筛查的普及，肺癌的疾病谱正在发生变化，以往以中央型局部晚期肺鳞癌为主，而目前肺癌疾病谱主要为以孤立性肺结节、磨玻璃结节、亚厘米结节等为影像学表现的早期肺腺癌。早期肺癌是目前临床研究的热点，本文将就早期肺癌外科治疗的多个方面进展进行述评。

## Abstract (en)

Lung cancer is the leading cause of cancer-related deaths worldwide, computed tomography screening has made the disease spectrum of lung cancer shift from the previously predominating central local advanced squamous cell carcinoma to early stage lung adenocarcinoma represented by solitary pulmonary nodule, ground-glass opacity (GGO) and sub-centimeter nodule. This paper reviewed the recent proceeding in the surgical management of early stage lung cancer.

肺癌是恶性肿瘤患者首位的死亡原因，全球每年新发肺癌患者180万，每年死于肺癌的患者约160万^[1]^。随着计算机断层扫描（computed tomography, CT）等影像筛查的普及，肺癌的疾病谱正在发生变化，以往以中央型局部晚期肺鳞癌为主，而目前肺癌疾病谱主要为以孤立性肺结节、磨玻璃结节、亚厘米结节等为影像学表现的早期肺腺癌^[2-4]^。因此，早期肺癌是目前临床研究的热点。

外科手术一直被认为是早期非小细胞肺癌（non-small cell lung cancer, NSCLC）治疗的金标准，近年来以立体定向放射治疗为代表的局部治疗方法正在挑战外科手术的绝对地位。2015年，Chang等^[5]^通过58例随机对照试验比较立体定向消融放射治疗（stereotactic ablative radiotherapy, SABR）和外科手术治疗Ⅰ期肺癌患者发现，SABR患者的3年总体生存和无复发生存不劣于外科手术患者，但研究样本量较少，随访期较短，同时局部放射治疗无法明确肺癌的准确淋巴结分期，局部放疗在短期内尚不能完全取代外科治疗，以往的传统外科治疗方式是否仍试用于目前的早期肺癌，还存在诸多疑问。因此，外科手术仍然是治疗早期肺癌的首选方式。传统的开放手术由于创伤大而渐被胸腔镜下肺切除术所取代。本文对胸腔镜下早期非小细胞肺癌切除术的进展进行综述。

## 1 切口的变化与争议

### 1.1 电视辅助胸腔镜手术（video-assisted thoracic surgery, VATS）

VATS创伤小，恢复快，长期生存效果与开放手术相似，目前已成为早期肺癌的标准外科手术方式^[6]^。VATS已演变出多种不同的手术方式，如切口数量从最初的三孔或四孔法，目前已发展为单孔法^[7, 8]^；切口位置也发生改变，创造出经剑突下VATS手术^[9]^；切口大小和位置的改变，新生了microlobectomy^[10]^；术中麻醉方式的改变，发展出了非插管VATS手术^[11]^。

2011年，西班牙Gonzalez医生^[8]^首先应用单孔VATS成功使用肺叶切除治疗早期肺癌，目前已成功应用单孔VATS开展外科治疗，包括早期肺癌的主要手术方式，如肺段切除，肺叶切除等，以及局部晚期肺癌的手术方式，如双肺叶切除、肺袖式切除、全肺切除、局部胸壁切除等。有研究^[7]^表明，单孔VATS可以有效减少切口疼痛，加快术后恢复，而且淋巴结清扫彻底程度并不劣于传统多孔法VATS。单孔VATS由于其与多孔法VATS的操作方式存在较大差别，器械间干扰较大，外科医生的操作舒适度较低，在一定程度上限制了其应用。随着单孔VATS器械的进步，其手术难度将进一步降低。

2015年，英国医生Joel Dunning^[10]^在传统三孔VATS的基础上进行了创新。肋间隙多为8 mm左右，因此将手术主操作切口从肋间移到了剑突下，同时将另外两个10 mm肋间切口改为为5 mm的肋间切口，用于放置5 mm的镜头和切割闭合器，Dunning医生将该手术方式命名为Microlobectomy，目前已成功开展了各个肺叶的切除。该手术方式可在不增加外科医生手术操作难度的前提下，尽量减少患者的创伤，符合微创外科的理念，但目前其微创效果仅存在于理论上，尚缺乏总结性临床研究数据发表，其安全性和有效性尚待进一步确定。

术中麻醉方式的改变催生了非插管VATS手术，即在清醒状态下行VATS手术，可避免全身麻醉下气管插管单肺机械通气，维持了正常生理状态，从而减少手术和麻醉给患者带来的损伤，加快术后康复^[11]^。陈晋兴和何建行已成功应用该手术方式治疗肺癌患者^[11, 12]^，该术式对外科医生的手术操作技巧有更高的要求，目前仍处于初步推广阶段。

### 1.2 机器人辅助肺切除术

近10年来，达芬奇机器人开始应用于肺癌外科，该系统具有三维立体放大视野，机械臂拥有7个自由度，可以模仿外科医生各种手术操作动作，使得手术操作更加灵活方便，目前有少数小样本研究对比了机器人与胸腔镜肺切除的手术效果。部分研究发现，机器人在手术出血量，操作时间方面均可与VATS类似，部分甚至优于VATS，同时淋巴结清扫范围也可以达到开放手术的效果^[13]^。

近年来，机器人技术在不断进步，已挑战VATS在早期肺癌外科治疗中的地位，如荧光显影技术帮助探查血管以及淋巴结^[14]^，集合超声探头用以术中准确定位肺部小结节。另一项创新是单孔机器人系统，可以使切口真正做到单孔^[15]^，同时不影响外科医生操作的舒适度，但这些技术尚缺乏在胸外科的应用，缺乏可靠的临床经验数据积累。机器人费用高昂，目前处于垄断地位，需要引入更多商业竞争以促使费用的下降。另外，目前的临床研究多为围手术期的短期研究，尚缺乏长期生存对比数据。

## 2 切除切除范围的变化与争议

1995年，肺癌研究组（Lung Cancer Study Group, LCSG）的随机对照试验比较了亚肺叶切除与肺叶切除治疗肺癌的生存效果，发现肺叶切除可以显著提高肺癌患者的长期生存^[16]^，同时降低术后局部复发率，肺叶切除被作为早期肺癌外科治疗的标准术式。由于疾病谱的改变，早期肺癌患者被广泛检出，肺叶切除的地位正在受到肺段切除等方式的挑战。有研究发现，早期肺癌，尤其是 < 2 cm的周围型肺癌使用肺段切除的远期生存与肺叶切除相似，同时可以显著提高患者术后生存质量^[17-20]^，但各项研究间结果不一致，最主要的争议是肺段切除的适应证选择。根据美国国立综合癌症网络（National Comprehensive Cancer Network, NCCN）指南，肺段切除适用于心肺功能不能耐受肺叶切除的患者，或者肿瘤≤2 cm且满足下列任意条件：①病理学类型为原位腺癌（adenocaminoma *in situ*, AIS）；②肺部毛玻璃样结节（ground-glass opacity, GGO）成分≥50%；③影像学监测提示倍增时间≥400 d，但该建议证据支持级别尚低，仍有待于正在开展的两项随机对照试验（CALGB 140503, JCOG0802）确定肺段切除的最佳适应证。肺段切除如能作为早期肺癌的标准治疗术式，除了肿瘤学效果，即生存效果，仍有其他诸多问题需要解决，如段间平面的准确确定、切缘距离、非靶段淋巴结转移、解剖畸形、术后并发症控制等。

针对处于多个肺段交界的极早期肺癌，目前有部分中心开展了亚肺段联合切除术，多是在3D-CT或3D打印模型评估后进行的S2b+S3a亚段联合切除术^[21, 22]^，说明随着对肺解剖认识的深入，目前的外科技术已经可以做到精准的亚肺段切除，尽管其在早期肺癌的临床价值尚不能确定。

## 3 淋巴结系统清扫与系统采样的争议

肺癌的淋巴结清扫方式多样，系统性淋巴结清扫一直是早期肺癌的标准方式，但系统性淋巴结清扫的并发症一直为外科医生诟病。如何优化淋巴结清扫，是目前肺癌外科研究的热点。有随机对照试验表明，对于术中采样确定无纵隔淋巴结转移的T1、T2期肺癌患者行纵隔淋巴结清扫与纵隔淋巴结采样远期生存效果相似^[23]^；另一方面，有临床回顾性分析显示，对于临床早期肺癌患者，术后病理检查证实存在淋巴结转移有着一定的比例，即隐匿性淋巴结转移。近年来，有多种淋巴结清扫优化方式被提出，一是根据肿瘤位置特异性淋巴结清扫，该清扫方式基于部分临床研究发现不同肺叶肺段位置的肿瘤存在不同的淋巴结转移途径^[24]^。有临床研究表明，肺叶特异性淋巴结清扫术后局部复发率与系统性淋巴结清扫类似^[25]^；另一种是根据淋巴结示踪技术确定需要清扫淋巴结的范围，实现精准化的淋巴结清扫，目前主要有术前^99m^Tc标记硫胶体、锡胶体淋巴结闪烁判定法，以及术中使用生物活性染料，如吲哚菁绿、放射性同位素法和近红外线法确定可能转移淋巴结，从而帮助优化淋巴结清扫的范围，但上述方法准确性尚低，且程序复杂，费用高昂，限制了其的大规模应用。

## 4 快速康复管理

在微创技术的推动下，快速康复外科理念在胸外科发展迅速。快速康复外科是指应用各种有效措施对手术患者进行干预，减少手术并发症，加速患者术后康复的方法。对于早期肺癌患者而言，在手术微创化的同时，术后管理也在进一步的流程微创化，其中比较引人注目的是术后胸管的管理。胸管是胸外科术后患者疼痛的主要来源，因此早期拔管、胸管改进，或者术后不常规放置胸管理论上可极大加快患者术后康复。以往胸管拔除指征为没有漏气且引流量少于200 mL/d。有研究^[26]^显示引流量为500 mL/d拔除胸管也并不增加胸腔积液风险。目前也有部分报道^[27, 28]^显示，对于早期肺癌楔形切除患者选择性的术后不常规放置胸管，未发现明显的需处理的并发症有升高趋势，不放置胸管将极大的加速患者康复及缩短患者住院时间。

## 5 总结

肺癌的疾病谱发生了巨大的变化，早期肺癌检出逐渐增多，外科手术仍是治疗早期非小细胞肺癌的主要方式，但以立体定向放射治疗为主的其他局部治疗方式正在逐渐向外科手术发起挑战，疾病谱的改变势必推动外科手术路径，手术切除范围等各方面的发展和改变，实现早期肺癌患者的精确化外科治疗。
